# Supplementary material for: Neural representation of emotional valence in human amygdala and surrounding regions
Source: Neuroimage. Author manuscript; Available in PMC 2026 Aug 4. (PMC13436427; doi:10.1016/j.neuroimage.2026.121693)
Supplement: 1 [file NIHMS2189230-supplement-1.docx]

## Supplementary Materials

### Head motion assessment

The current dataset contains 20 participants who were scanned in two time periods. The first 11 were recorded in 2010, when 15 subjects were scanned and 4 were excluded due to excessive head motion. A manuscript was published on the data from the 11 subjects (Liu et al., Neural Substrate of the Late Positive Potential in Emotional Processing, *Journal of Neuroscience* 32(42):14563–14572, 2012). The remaining 9 participants were recorded in 2017 when 11 participants gave informed consent but 2 withdrew before scanning. The 2017 recordings used the same stimuli, the same experimental paradigm, the same scanner, and the same scanning parameters as the 2010 recordings.

The researcher who conducted the 2010 recordings and performed subsequent preprocessing has since left academia. No record of the reject data (N=4) was kept. We are thus not able to provide the exact motion threshold used for exclusion. No subjects were excluded from the 2017 recordings.

For the 20 subjects analyzed in this paper, we assessed head motion using framewise displacement (FD). As shown in Figure S1, none of the participants exhibited average FD greater than 0.5 mm.


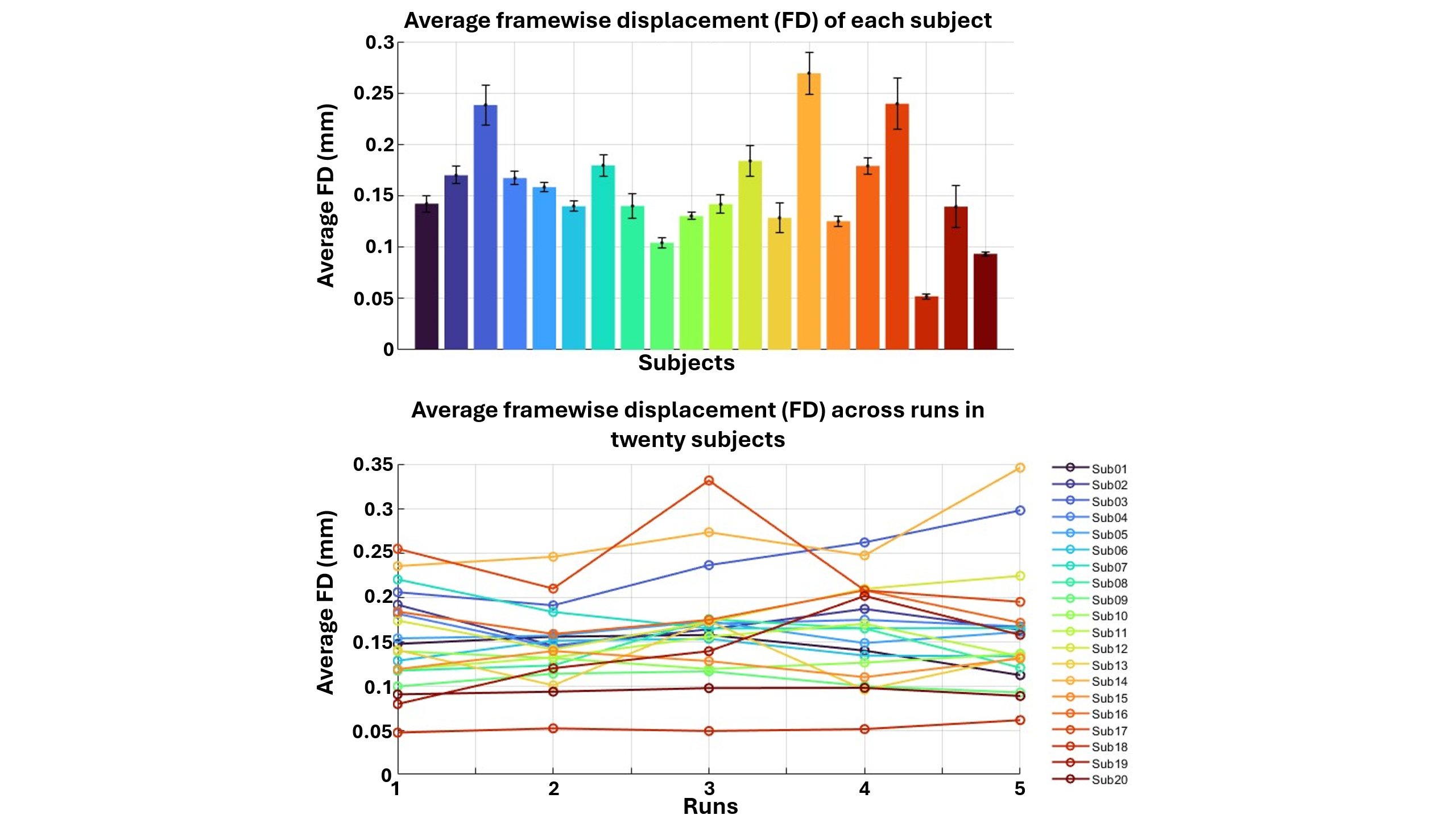


**Figure S1.** Head motion assessment of the N=20 subjects included in this study. **(A)** Average framewise displacement of each subject. **(B)** Average framewise displacement of each subject in each run.

### IAPS picture complexity and valence

We examined whether perceived complexity and mathematical complexity quantified by entropy impacted valence ratings. As shown in Figure S2, there were no significant relationships: complexity–valence correlation (R = -0.057, P = 0.67) and entropy–valence correlation (R = -0.068, P = 0.6).

*
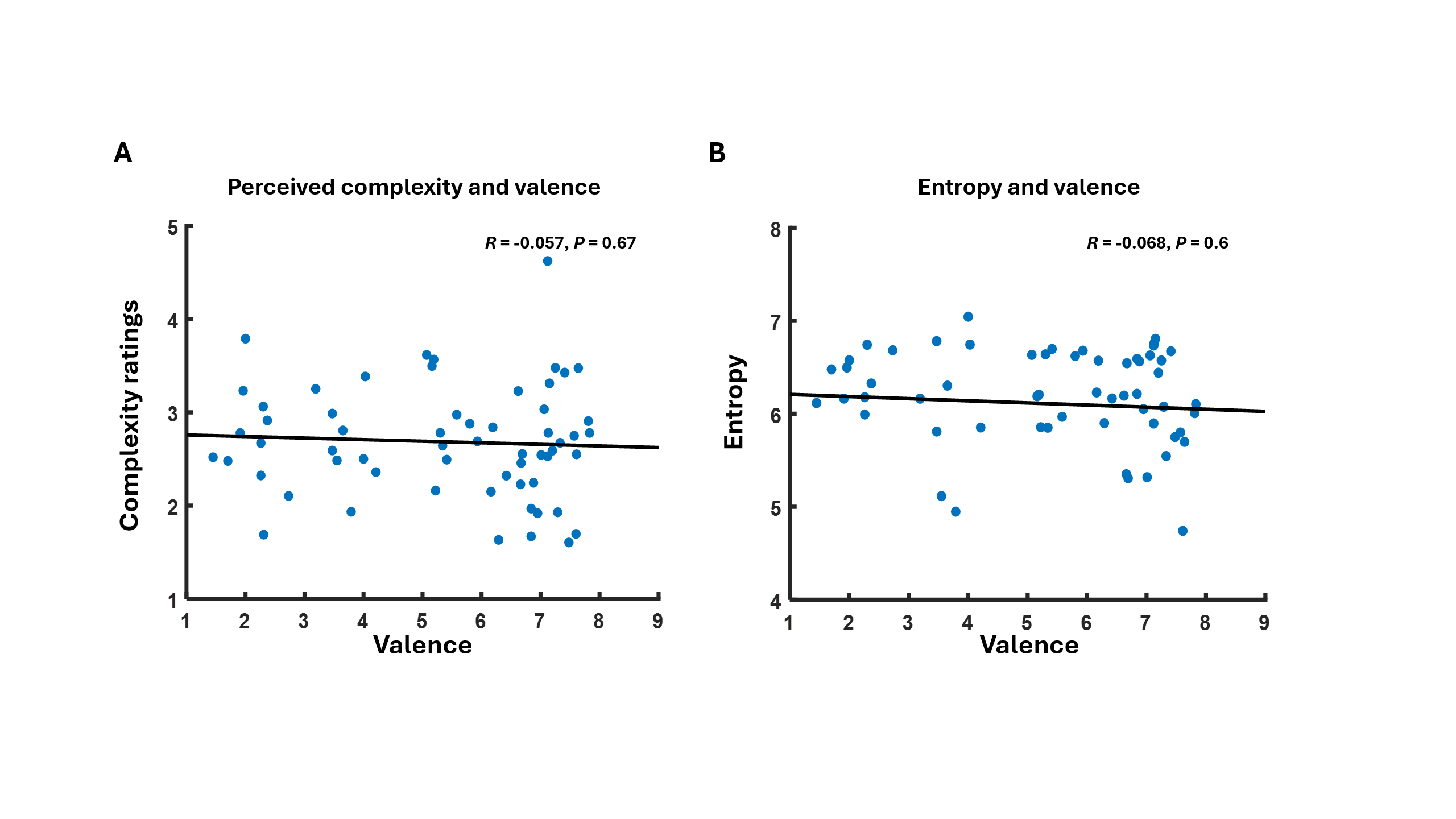
*

**Figure S2.** Figure R5. IAPS picture complexity and valence. **(A)** Correlation between perceived complexity ratings and valence for IAPS pictures. **(B)** Correlation between entropy and valence for IAPS pictures.

### Analysis in subnuclei of amygdala

Three amygdala subregions (lateral nucleus, basolateral nucleus, and cortical/medial nuclei), each containing at least 10 voxels, are considered. As shown in Figure S3, we found that both univariate and multivariate regression models significantly predicted valence in the lateral amygdala (R(Univariate) = 0.146, p = 0.001; R(Multivariate) = 0.155 , p = 0.001) and basolateral amygdala (R(Univariate) = 0.158, p < 0.001; R(Multivariate) = 0.166 , p < 0.001); here the p-value was obtained by comparing with chance level in the random permutation distribution. In the cortical/medial amygdala, however, only the univariate model demonstrated significant predictive performance (R(Univariate) = 0.077, p = 0.007; R(Multivariate) = 0.047, p = 0.142). Importantly, consistent with the findings from the whole amygdala analysis, there were no significant differences between univariate and multivariate regression performance within any of the three subdivisions (p > 0.36).


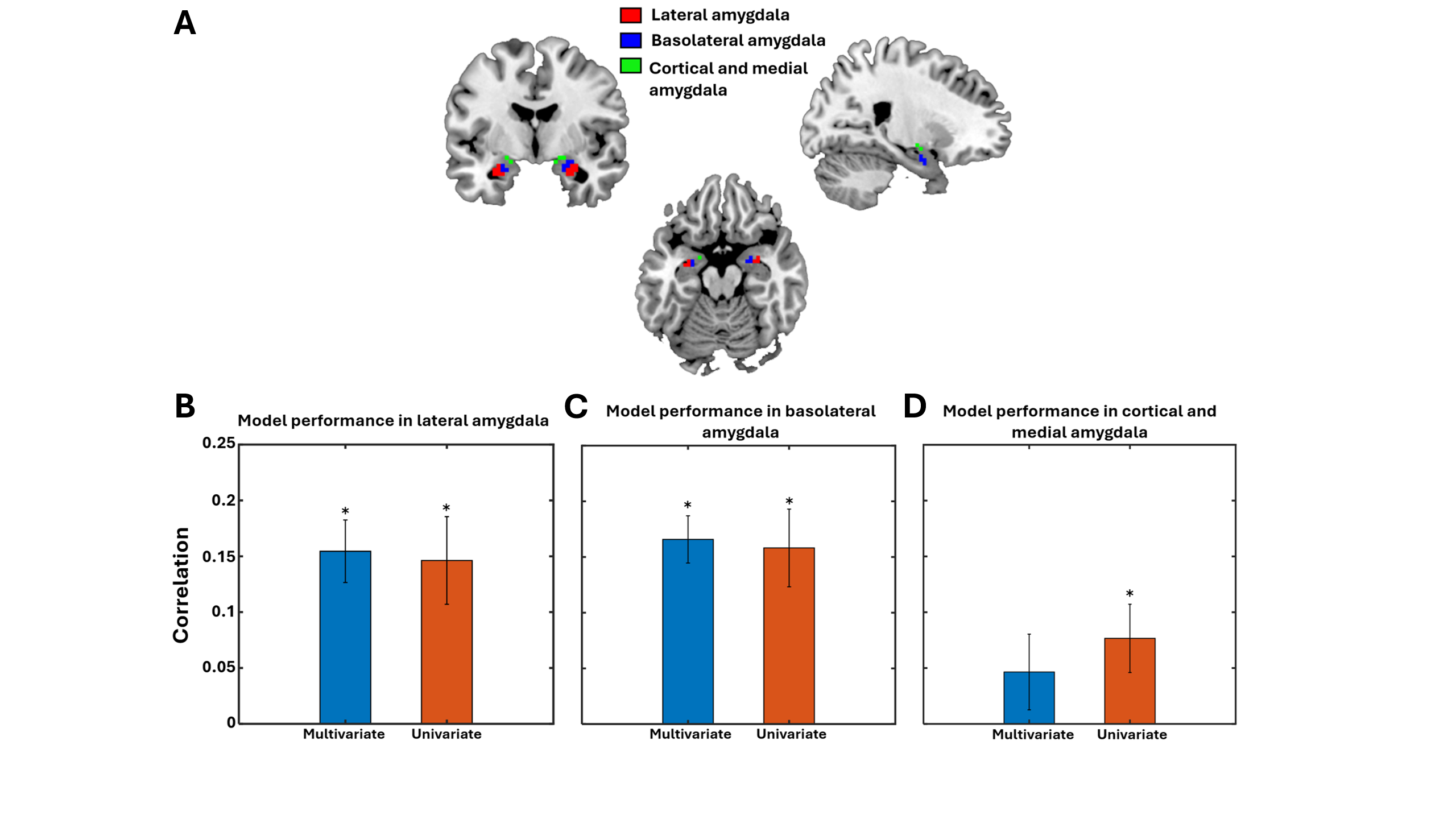


**Figure S3.** Valence representation in subregions of the amygdala. **(A)** Visualization of three subnuclei of amygdala in this analysis. **(B)** Comparison of the performance of the multivariate regression vs the univariate regression models in lateral amygdala. **(C)** Comparison of the performance of the multivariate regression vs the univariate regression models in basolateral amygdala. **(D)** Comparison of the performance of the multivariate regression vs the univariate regression models in cortical and medial amygdala.

### Regression analysis after arousal regress-out

To examine whether arousal potentially influenced our valence representation analysis, we did a control analysis in which arousal is regressed out from the valence ratings and the brain responses and the same univariate and multivariate regression analyses were carried out on the residuals. Similar results were obtained (Figure S4). In particular, consistent with our main analysis, the difference between the multivariate model and the univariate model became significant only when the core amygdala mask was expanded to include larger surrounding regions.


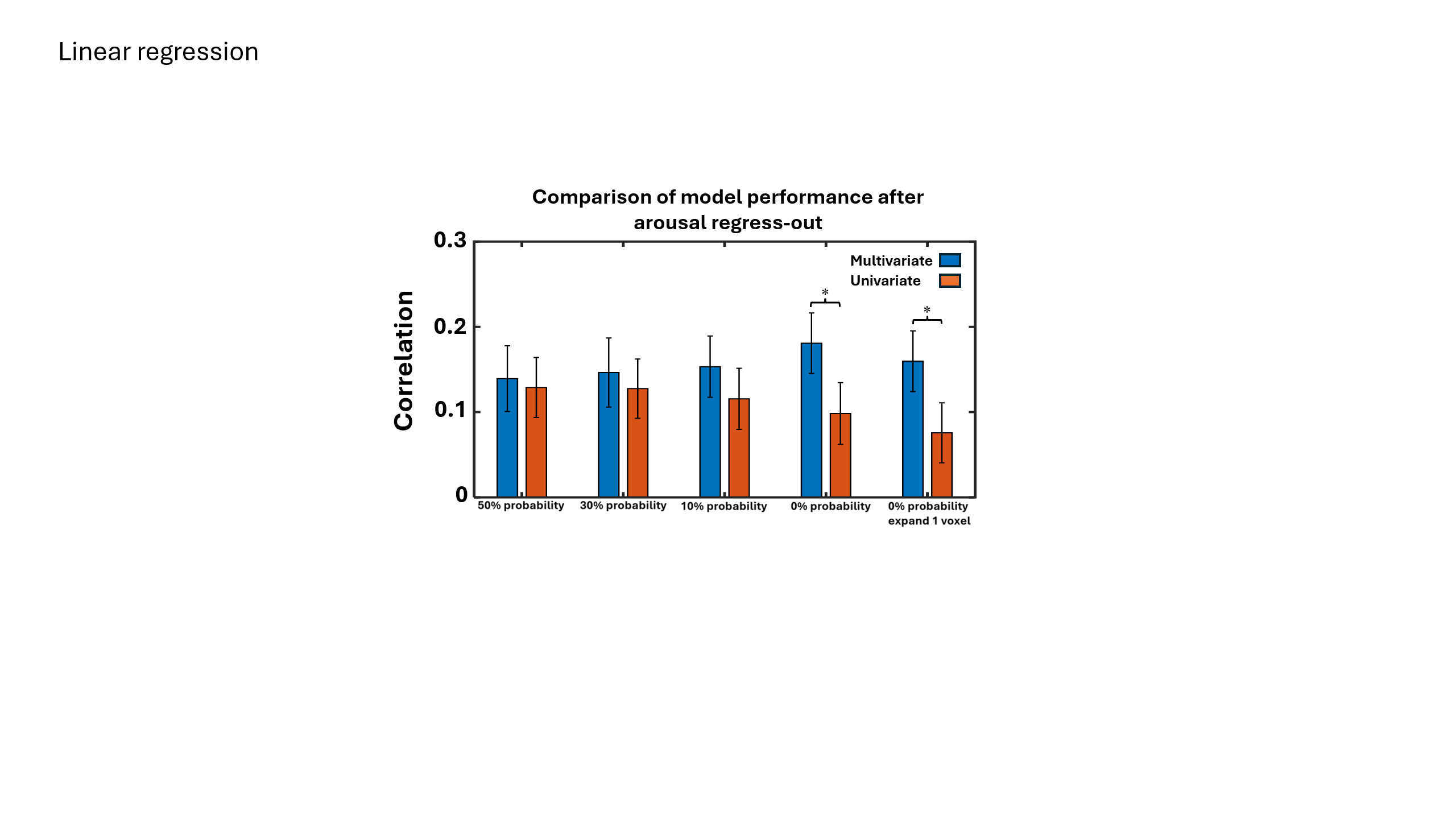


**Figure S4.** Comparison of model performance in predicting valence after regressing out arousal.

### GLM analysis in finer temporal windows

To examine whether the amygdala’s response may differ between early and late time periods, we conducted an additional temporal analysis based on estimating early and late amygdala responses separately. Specifically, we regenerated the beta series estimates using a shorter event duration by splitting the original regressor into two (early and late; each 1.5 s), and early and late amygdala responses were then used to fit the univariate and multivariate linear regression models in both the core amygdala and the three parcellated subdivisions. The results are shown below.

See Figure S5. The statistics are as follows: core amygdala early phase: R (M) = 0.168, p < 0.001 , R (U) = 0.152, p = 0.001; core amygdala late phase: R (M) = 0.134, p < 0.001 , R (U) = 0.15, p = 0.005; lateral amygdala early phase: R (M) = 0.136, p = 0.004, R (U) = 0.142, p = 0.002; lateral amygdala late phase: R (M) = 0.147, p = 0.001, R (U) = 0.14, p = 0.003; basolateral amygdala early phase: R (M) = 0.149, p < 0.001, R (U) = 0.153, p = 0.001; basolateral amygdala late phase: R (M) = 0.157, p = 0.003, R (U) = 0.151, p < 0.001; cortical and medial amygdala early phase: R (M) = 0.041, p = 0.523, R (U) = 0.084, p = 0.005; cortical and medial amygdala late phase: R (M) = 0.047, p = 0.162, R (U) = 0.062, p = 0.056; here U=Univariate and M=Multivariate. In the whole amygdala, as well as within the subdivisions lateral and basolateral amygdala, both univariate and multivariate models significantly predicted valence based on both early and late responses. In contrast, only the univariate model significantly predicted valence from early responses in the cortical and medial amygdala. Importantly, consistent with our primary analysis, there were no significant differences in regression performance between univariate and multivariate approaches for either early or late responses across the core amygdala or any of the subdivisions.


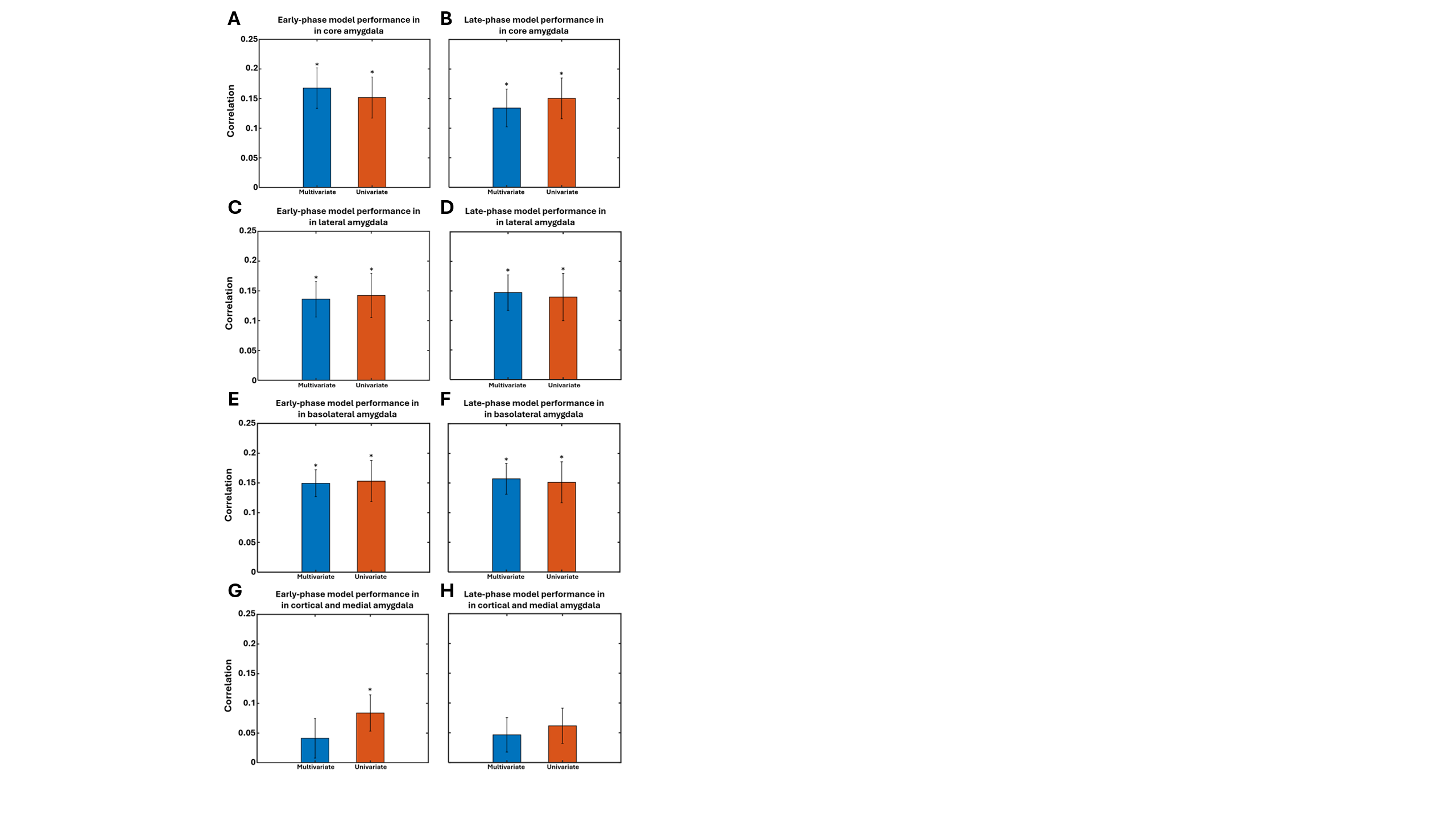


**Figure S5.** Valence prediction from early and late responses. **(A)** Comparison of the performance of the multivariate regression vs the univariate regression models in core amygdala during early-phase. **(B)** Comparison of the performance of the multivariate regression vs the univariate regression models in core amygdala during late-phase. **(C)** Comparison of the performance of the multivariate regression vs the univariate regression models in lateral amygdala during early-phase. **(D)** Comparison of the performance of the multivariate regression vs the univariate regression models in lateral amygdala during late-phase. **(E)** Comparison of the performance of the multivariate regression vs the univariate regression models in basolateral amygdala during early-phase. **(F)** Comparison of the performance of the multivariate regression vs the univariate regression models in basolateral amygdala during late-phase. **(G)** Comparison of the performance of the multivariate regression vs the univariate regression models in cortical and medial amygdala during early-phase. **(H)** Comparison of the performance of the multivariate regression vs the univariate regression models in cortical and medial amygdala during late-phase.

### Additional information on the expanded amygdala ROIs

Additional information on the expanded amygdala ROIs is described in Table S1.

**Table S1. Further information on core amygdala and each expanded ROI.**

|  | ROI | Side | Number of voxels | Center coordinate |
| --- | --- | --- | --- | --- |
|  | Core amygdala | Left | 47 | -24, -1, -23 |
|  | Core amygdala | Right | 42 | 21, -1, -23 |
|  | 30% probability only | Left | 72 | -24, -1, -20 |
|  | 30% probability only | Right | 78 | 21, -1, -20 |
|  | 10% probability only | Left | 63 | -24, -1, -20 |
|  | 10% probability only | Right | 69 | 21, -1, -20 |
|  | 0% probability only | Left | 130 | -24, -4, -20 |
|  | 0% probability only | Right | 116 | 21, -1, -20 |
|  | 0% probability + 1 voxel expansion only | Left | 229 | -24, -4, -20 |
|  | 0% probability + 1 voxel expansion only | Right | 228 | 21, -1, -20 |

***References***

Glasser, M. F., Coalson, T. S., Robinson, E. C., Hacker, C. D., Harwell, J., Yacoub, E., ... & Van Essen, D. C. (2016). A multi-modal parcellation of human cerebral cortex. *Nature*, 536(7615), 171-178.
